# The IL1β-IL1R signaling is involved in the stimulatory effects triggered by hypoxia in breast cancer cells and cancer-associated fibroblasts (CAFs)

**DOI:** 10.1186/s13046-020-01667-y

**Published:** 2020-08-10

**Authors:** Rosamaria Lappano, Marianna Talia, Francesca Cirillo, Damiano Cosimo Rigiracciolo, Domenica Scordamaglia, Rita Guzzi, Anna Maria Miglietta, Ernestina Marianna De Francesco, Antonino Belfiore, Andrew H. Sims, Marcello Maggiolini

**Affiliations:** 1 Department of Pharmacy, Health and Nutritional Sciences, University of Calabria, 87036 Rende, Italy; 2 Department of Physics, University of Calabria, 87036 Rende, Italy; 3 Regional Hospital Cosenza, 87100 Cosenza, Italy; 4 Endocrinology, Department of Clinical and Experimental Medicine, University of Catania, Garibaldi-Nesima Hospital, 95122 Catania, Italy; 5 MRC Institute of Genetics and Molecular Medicine, University of Edinburgh, Edinburgh, EH4 2XR UK

**Keywords:** Hypoxia, Hypoxia inducible factor-1α (HIF-1α), Interleukin-1β (IL-β), G protein estrogen receptor (GPER), Breast cancer, Cancer-associated fibroblasts (CAFs)

## Abstract

**Background:**

Hypoxia plays a relevant role in tumor-related inflammation toward the metastatic spread and cancer aggressiveness. The pro-inflammatory cytokine interleukin-1β (IL-β) and its cognate receptor IL1R1 contribute to the initiation and progression of breast cancer determining pro-tumorigenic inflammatory responses. The transcriptional target of the hypoxia inducible factor-1α (HIF-1α) namely the G protein estrogen receptor (GPER) mediates a feedforward loop coupling IL-1β induction by breast cancer-associated fibroblasts (CAFs) to IL1R1 expression by breast cancer cells toward the regulation of target genes and relevant biological responses.

**Methods:**

In order to ascertain the correlation of IL-β with HIF-1α and further hypoxia-related genes in triple-negative breast cancer (TNBC) patients, a bioinformatics analysis was performed using the information provided by The Invasive Breast Cancer Cohort of The Cancer Genome Atlas (TCGA) project and Molecular Taxonomy of Breast Cancer International Consortium (METABRIC) datasets. Gene expression correlation, statistical analysis and gene set enrichment analysis (GSEA) were carried out with R studio packages. Pathway enrichment analysis was evaluated with Kyoto Encyclopedia of Genes and Genomes (KEGG) pathway. TNBC cells and primary CAFs were used as model system. The molecular mechanisms implicated in the regulation of IL-1β by hypoxia toward a metastatic gene expression profile and invasive properties were assessed performing gene and protein expression studies, PCR arrays, gene silencing and immunofluorescence analysis, co-immunoprecipitation and ChiP assays, ELISA, cell spreading, invasion and spheroid formation.

**Results:**

We first determined that IL-1β expression correlates with the levels of HIF-1α as well as with a hypoxia-related gene signature in TNBC patients. Next, we demonstrated that hypoxia triggers a functional liaison among HIF-1α, GPER and the IL-1β/IL1R1 signaling toward a metastatic gene signature and a feed-forward loop of IL-1β that leads to proliferative and invasive responses in TNBC cells. Furthermore, we found that the IL-1β released in the conditioned medium of TNBC cells exposed to hypoxic conditions promotes an invasive phenotype of CAFs.

**Conclusions:**

Our data shed new light on the role of hypoxia in the activation of the IL-1β/IL1R1 signaling, which in turn triggers aggressive features in both TNBC cells and CAFs. Hence, our findings provide novel evidence regarding the mechanisms through which the hypoxic tumor microenvironment may contribute to breast cancer progression and suggest further targets useful in more comprehensive therapeutic strategies.

## Background

The triple-negative breast cancer (TNBC), which accounts for 10–20% of all breast malignancies, is characterized by the absence of the estrogen receptor (ER) and the progesterone receptor (PR) without the amplification of the human epidermal growth factor receptor 2 (HER2) [1]. TNBC displays a high heterogeneity, aggressive features and poor overall survival rates [1–3]. Targeted therapeutics for TNBC are currently limited and the chemotherapy still remains the mainstay of treatment, though the patients frequently develop resistance [4]. Therefore, the identification of novel targeted therapies is an imperative challenge to substantially improve the outcome of the TNBC patients. In this context, hypoxia and related effectors, as the hypoxia-inducible factors, have been suggested as important hallmarks of TNBC [5, 6]. To date, hypoxia that represents one of the most common conditions encountered within the breast cancer microenvironment, has been associated with tumor progression, increased risk of metastasis and mortality [7]. Malignant cells may adapt to hypoxia mainly through the action of the hypoxia-inducible factor-1α (HIF-1α) [8], which has been largely involved in tumor growth and vascularization, stromal cell recruitment, extracellular matrix remodeling, premetastatic niche formation, invasion and metastasis [7]. In this regard, it is worth mentioning that an elevated expression of HIF-1α has been associated with the aggressiveness of breast cancer and poor clinical outcomes [9]. Likewise, the over-expression of HIF-1α and the consequent hyperactivation of its target genes have been indicated as key drivers in the TNBC [10, 11].

Numerous transduction pathways, including the G protein-coupled receptors (GPCRs), are engaged by the hypoxia-mediated activation of the HIF-1α signaling [12]. In this regard, our previous studies have demonstrated that upon hypoxia and other stimulatory factors, the G protein estrogen receptor, namely GPER, may contribute to the action of HIF-1α toward breast tumor growth and angiogenesis [13–17]. For instance, HIF-1α was shown to be required for the up-regulation of GPER by hypoxia and both HIF-1α and GPER triggered the up-regulation of the vascular endothelial growth factor (VEGF) in breast cancer cells and main components of the tumor microenvironment, as cancer-associated fibroblasts (CAFs) [13].

The breast cancer microenvironment is characterized by an intricate functional interplay among epithelial cancer cells, the surrounding non-cancerous stromal cells (i.e. CAFs, adipocytes, endothelial, mesenchymal, innate and adaptive immune cells) and non-cellular components (i.e. blood and lymphatic vessels, extracellular matrix components) [18]. For instance, the bi-directional and intimate communication of cancer cells with CAFs has been reported to contribute to worse outcomes. In this vein, it is well acknowledged that cancer cells through various factors educate fibroblasts toward their pro-tumorigenic changes, which then characterize the stimulatory action of CAFs [18–20]. Indeed, CAFs sustain the malignant properties of cancer cells synthesizing diverse extracellular matrix components (ECM), cytokines and growth factors, which in turn contribute to the metastatic cancer progression [21]. In this scenario, the dynamic network occurring between cancer cells and CAFs relies on the production of many effectors, as diverse chemokines exerting tumor-promoting effects [22].

The interleukin-1 (IL-1) family consists of several agonists, including IL-1α and IL-1β, and three receptor antagonists including the specific receptor antagonist IL1R1a [23]. The IL-1 receptor (IL-1R) family is instead composed of more than 10 members, including the two main receptors namely IL-1 receptor type I (IL1R1) and the decoy receptor IL-1R type II [23]. As it concerns IL-1β, its involvement in metastatic and angiogenic pathways toward breast cancer progression has been reported [24, 25]. In this regard, our and other previous studies have demonstrated that an estrogen-induced feedforward loop couples the IL-1β induction in CAFs to the IL1R1-stimulatory action elicited in breast cancer cells [26]. Likewise, high IL1β levels within the breast tumor microenvironment have been associated with a high tumor grade and an invasive cancer phenotype [27].

In the framework of the aforementioned data, here we first show that a positive correlation between HIF-1α and IL-1β expression occurs in large cohorts of TNBC patients, as ascertained by querying public datasets. Thereafter, we provide novel mechanistic evidence on the hypoxia-prompted liaison between the HIF-1α/GPER signaling and the IL-1β/IL1R1 axis, toward a metastatic gene expression profile of TNBC cells and the engagement of CAFs in facilitating invasive cancer features.

## Methods

### Publicly available molecular datasets

Gene expression analysis was performed using the publically available The Cancer Genome Atlas (TCGA) and Molecular Taxonomy of Breast Cancer International Consortium (METABRIC) datasets [28, 29]. Data was downloaded on the 10 May 2020. The patients clinical information along with the mRNA expression data (RNA Seq V2 RSEM) reported in the Invasive Breast Cancer Cohort of the TCGA project were downloaded from UCSC Xena (https://xenabrowser.net/). The clinical information and the microarray gene expression data (Log2 transformed intensity values) of the METABRIC cohort (n. 2509) were retrieved from cBioPortal for Cancer Genomics (http://www.cbioportal.org/). Samples of the TCGA cohort (n. 1247) were filtered by the “sample type” in order to obtain exclusively the tumor tissues (n. 1101). Thereafter, patients of both TCGA and METABRIC were classified on the basis of the presence or the absence of the estrogen receptor (ER), progesterone receptor (PR) and human epidermal growth factor receptor 2 (HER2), detected by immunohistochemistry. Gene expression and clinical information were also filtered for missing values. The final filtering resulted in 771 patients of TCGA and 1904 patients of METABRIC.

### Correlation and pathway enrichment analysis

The Pearson correlation coefficients (r-values) between the mRNA levels of HIF-1α and the other genes of the TCGA (n. 20,530) dataset in TNBC cohort of patients were assessed in R Studio (version 3.6.1) using the *cor.test()* function and setting the method as “Pearson”. The statistical analysis was performed by using the t-tests considering significant the coefficients obtained with *p* < 0.001. The first 250 most correlated genes were selected for the next evaluations. In particular, aiming to cluster these genes in pathways, we uploaded our list on the Database for Annotation, Visualization and Integrated Discovery (DAVID) functional annotation analysis website (https://david.ncifcrf.gov/). We analyzed the genes selecting the functional annotation tool and the option Kyoto Encyclopedia of Genes and Genomes (KEGG) pathways, choosing the official gene symbol as “select identifier” and gene list as “list type” in the options of the upload and selecting a limit species of “*Homo sapiens*” in the background.

### Gene set enrichment analysis (GSEA)

Gene Set Enrichment Analysis (GSEA) was performed using the *gsea()* function of the *phenoTest* package (https://bioconductor.org/packages/release/bioc/html/phenoTest.html) in R Studio. The KEGG “Cytokine-cytokine receptor interaction” pathway (KEGG entry = hsa04060) and “HIF-1 signaling” pathway (KEGG entry = hsa04066) were selected as the reference gene sets. We ranked the genes of the “Cytokine-cytokine receptor interaction” pathway in accordance with the differential expression within HIF-1α high and low (median expression value as threshold assessment) samples, and the “HIF-1 signaling” pathway genes in accordance with the differential expression within IL-1β high and low (median expression value as threshold assessment) samples. Both analysis were performed only in the TNBC subgroup of patients, verifying if the selected sets of genes were enriched at the bottom or the top of the ranked lists. We calculated the enrichment score (ES) that reflects the degree to which a set of genes is overrepresented at the extremes (top or bottom) of the entire ranked list. The score was calculated by walking down a list of genes ranked by their correlation with the selected phenotype (high or low HIF-1α/ IL-1β levels), increasing a running-sum statistic when a gene in that gene set is encountered (each vertical line underneath the enrichment plot) and decreasing it when a gene that isn’t in the gene set is encountered. The magnitude of the increment depends on the correlation of one gene with the phenotype. In this analysis, 20,000 simulations were used (B = 20,000). *p* < 0.05 was considered significant.

### Reagents

The ROS scavenger N-acetyl-L-cysteine (NAC) (used at a 300 μM concentration) and the proteasome inhibitor MG132 (used at a 10 μM concentration) were purchased from Merck Life Science (Milan, Italy). PD98059 (PD) and LY294,002 (LY) (both used at a 1 μM concentration) were obtained from Calbiochem (Milan, Italy). All compounds were dissolved in DMSO, except NAC that was solubilized in water. Recombinant human IL-1β (used at a 10 ng/mL concentration) was purchased from Thermo Fisher Scientific (Life Technologies Italia, Monza, Italy) and solubilized in PBS with 1% BSA. The IL1R1 antagonist (IL1R1a) human recombinant protein (used at a 50 ng/mL concentration) was purchased from Thermo Fisher Scientific and solubilized in 20 mM TBS, pH 8, with 50% glycerol. Anti-IL-1β neutralizing antibody (MAB601) was purchased from R&D Systems (Bio-Techne, Milano, Italy).

### Cell cultures

The TNBC MDA-MB 231 breast cancer cells were provided by ATCC (Manassas, VA, USA), used less than 6 months after resuscitation, routinely tested and authenticated according to the ATCC suggestions. MDA-MB 231 cells were maintained in DMEM/F12 (Dulbecco’s modified Eagle’s medium) with phenol red, supplemented with 5% fetal bovine serum (FBS) and 1% penicillin/streptomycin (Thermo Fisher Scientific). CAFs were isolated, cultured and characterized as previously described [30] from 10 invasive mammary ductal carcinomas and pooled for the subsequent studies. Briefly, specimens were cut into 1–2 mm diameter pieces, placed in a digestion solution (400 IU collagenase, 100 IU hyaluronidase, 10% FBS, antibiotics and antimycotics) (Thermo Fisher Scientific) and incubated overnight at 37 °C. Cells were then separated by differential centrifugation at 90×g for 2 min. The supernatant containing fibroblasts were centrifuged at 485×g for 8 min, the pellet obtained was suspended in fibroblasts growth medium (Medium 199 and Ham’s F12 mixed 1:1 and supplemented with 10% FBS and 1% penicillin) (Thermo Fisher Scientific) and cultured at 37 °C, 5% CO2. CAFs were then expanded into 10-cm Petri dishes and stored as cells passaged for three population doublings within total 7 to 10 days after tis-sue dissociation. Primary cell cultures of fibroblasts were characterized by immunofluorescence with human anti-vimentin (V9; 1:500) and human anti-cytokeratin 14 (LL001) (Santa Cruz Biotechnology, DBA, Milan, Italy; 1:250). FAPα antibody (H-56; Santa Cruz Biotechnology, DBA, Milan, Italy; 1:500) was used to assess fibroblast activation (data not shown). We used CAFs passaged for up to 10 population doublings for the experiments, to minimize clonal selection and culture stress, which could occur during extended tissue culture. All cell lines were grown in a 37 °C incubator with 5% CO2 and switched to medium without serum and phenol red the day before treatments to be processed for immunoblot and RT-PCR assays.

### Gene expression studies and PCR arrays

Total RNA was extracted, and cDNA was synthesized by reverse transcription as previously described [31]. The expression of selected genes was quantified by real-time PCR using platform Quant Studio7 Flex Real-Time PCR System (Thermo Fisher Scientific). Gene-specific primers were designed using Primer Express version 2.0 software (Applied Biosystems) and are as follows: 5′-ACCTATGACCTGCTTGGTGC-3′ (HIF-1α forward) and 5′-GGCTGTGTCGACTGAGGAAA-3′ (HIF-1α reverse); 5′-TTAAAGCCCGCCTGACAGA-3′ (IL-1β forward) and 5′-GCGAATGACAGAGGGTTTCTTAG-3′ (IL-1β reverse); 5′-TTACAGAGGGAAAACGACACCT-3′ (GPER forward) and 5′-GTGGGTCTTCCTCAGAAGGG-3′ (GPER reverse); 5′-AGTCCCTGAGCATCTACGGT-3′ (COX2 forward) and 5′-CATCATCAGACCAGGCACCA-3′ (COX2 reverse); 5′-AAGCCACCCCACTTCTCTCTAA-3′ (ACTB forward) and 5′-CACCTCCCCTGTGTGGACTT-3′ (ACTB reverse). Assays were performed in triplicate and the results were normalized for actin beta (ACTB) expression and then calculated as fold induction of RNA expression.

PCR arrays were performed using a TaqMan™ Human Tumor Metastasis Array (Thermo Fisher Scientific) according to the manufacturer’s instructions. The amplification reaction and the results analysis were carried out using platform Quant Studio7 Flex Real-Time PCR System (Thermo Fisher Scientific).

### Gene silencing experiments and plasmids

Cells were transfected using X-treme GENE 9 DNA Transfection Reagent (Roche Diagnostics, Merck Life Science) for 24 h before treatments with a control vector and a specific shRNA sequence or antisense vector for each target gene. The short hairpin (sh) RNA constructs to knock down the expression of HIF-1α and the control shRNA construct were purchased form SABioscience Corporation. Antisense vector for GPER (AsGPER), which was generated by cloning of the entire open reading frame of the receptor cDNA in the reverse orientation in pcDNA3.1 Hygro (−), was a kind gift from Eric R Prossnitz (University of New Mexico Health Science Center, Albuquerque, USA). The plasmid DN/c-fos, which encodes for c-fos mutant that heterodimerizes with c-fos dimerization partners but does not allow DNA binding, was a kind gift from Dr. C. Vinson (NIH, Bethesda, MD, USA).

### Chromatin Immunoprecipitation (ChIP) assay

Cells were grown in 10-cm dishes, exposed to treatments for 16 h, and then cross-linked with 1% formaldehyde and sonicated. Supernatants were immuno-cleared with salmon DNA/protein A-agarose (Merck Life Science) and immunoprecipitated with anti-HIF-1α or anti-GPER antibody or nonspecific IgG. Pellets were washed, eluted with a buffer consisting of 1%SDS and 0.1 mol/L NaHCO3, and digested with proteinase K. DNA was obtained by phenol/chloroform extractions and precipitated with ethanol. The yield of target region DNA in each sample after ChIP was analyzed by real-time PCR. The primers used to amplify a region containing a HRE site located into the GPER promoter sequence were: 5′-TGCAGCACTTCAAAACAATAACC − 3′ (Fw) and 5′-GGGTTTGAGTTGTTTTTCCTTTGG-3′ (Rv); the primers used to amplify a region containing a HRE site located into the IL-1β promoter sequence were: 5′-ACAGACAGGGAGGGCTATTG-3′ (Fw) and 5′-GGGCAAGGAGTAGCAAACTA-3′ (Rv). Data were normalized to the input for the immunoprecipitation and the results were reported as fold changes respect to nonspecific IgG.

### Western blot analysis

Cells were grown in 10-cm dishes, exposed to treatments, and then lysed as previously described [32]. Equal amounts of whole-protein extract were resolved on a 10% SDS-polyacrylamide gel and transferred to a nitrocellulose membrane (Amersham Biosciences, Sigma-Adrich, Milan, Italy), which were probed with primary antibodies (1:1000) against HIF-1α and IL-1β (R&D Systems, Bio-Techne, Milano, Italy), GPER (AB137479) (Abcam, DBA, Milan, Italy), phosphorylated ERK1/2 (E-4), ERK2 (C-14), p-AKT1/2/3 (Ser 473)-R, AKT/1/2/3 (H-136), c-fos (E-8) and β-actin (AC-15; 1:4000) (Santa Cruz Biotechnology, DBA, Milan, Italy) and then revealed using the chemiluminescent substrate for western blotting Westar Nova 2.0 (Cyanagen, Biogenerica, Catania, Italy). For nuclear extracts, cells were lysed using 300 μl of cytosolic buffer (50 mM HEPES pH 7.5, 150 mM NaCl, 1% Triton X-100, 1.5 mM MgCl2, 1 mM EGTA, pH 7.5, 10% glycerol) with protease inhibitors (1.7 mg/ml aprotinin, 1 mg/ml leupeptin, 200 mmol/liter phenylmethylsulfonyl fluoride, 200 mmol/liter sodium orthovanadate and 100 mmol/liter sodium fluoride). Following centrifugation (14,000 g, 4 °C, 10 min), the supernatant was referred to as cytoplasmic fraction and the pellet containing nuclei was resuspended in high salt buffer (20 mM HEPES pH 7.9, 25% [v:v] glycerol, 420 mM NaCl, 1.5 mM MgCl2, 0.2 mM EDTA and protease inhibitors). For the extraction of nuclear proteins, the obtained solution was vortexed thoroughly, incubated overnight with agitation and centrifugated at 14000 g, 4 °C for 10 min. Equal amounts of the collected supernatant, which represent the nuclear fraction, were then run on 10% SDS-PAGE and western blot analysis was performed as described above. The purity of the nuclear fraction was confirmed by immunoblotting with primary antibodies against β-actin (AC-15; 1:4000) and anti-LMNB/Lamin (M-20; 1:2000) (Santa Cruz Biotechnology, DBA, Milan, Italy).

### Co-immunoprecipitation assay

After exposure to treatments, cells were washed and lysed using 500 μl RIPA buffer with protease inhibitors (1.7 mg/ml aprotinin, 1 mg/ml leupeptin, 200 mmol/liter phenylmethylsulfonyl fluoride, 200 mmol/liter sodium orthovanadate and 100 mmol/liter sodium fluoride). Samples were then centrifuged at 13,000 rpm for 10 min and protein concentrations were determined using Coomassie (Bradford) protein assay. Proteins (200 μg) were then incubated for 2 h with 900 μl of immunoprecipitation buffer with inhibitors, 2 μg of anti-c-fos or anti-GPER antibodies and 20 μl of Protein A/G agarose immunoprecipitation reagent (Santa Cruz Biotechnology, DBA, Milan, Italy). Samples were then centrifuged at 13,000 rpm for 5 min at 4 °C to pellet beads. Pellets were washed four times with 500 μl of PBS and centrifuged at 13,000 rpm for 5 min at 4 °C. Supernatants were collected, resuspended in 20 μl RIPA buffer with protease inhibitors, 2X SDS sample buffer and heated to 95 °C for 5 min. Samples were then run on 10% SDS-PAGE, transferred to nitrocellulose and probed with primary antibodies. Western blot analysis and ECL detection were performed as described above.

### Immunofluorescence microscopy

Cells were grown on a cover slip, serum deprived for 18 h and then exposed to treatments for 16 h. Next, cells were fixed in 4% paraformaldehyde in PBS, permeabilized with 0.2% Triton X-100, washed 3 times with PBS and incubated at 4 °C overnight with a primary antibody (1:250) against GPER (AB137479) (Abcam, DBA, Milan, Italy) or Phospho-Myosin Light Chain 2 (Ser19) (p-MLC) (Cell Signaling, Euroclone, Milan, Italy). After incubation, the slides were extensively washed with PBS, probed with alexa Fluor 594 goat anti-rabbit IgG (1:300, Thermo Fisher Scientific) and 4′,6-diamidino-2-phenylindole dihydrochloride (DAPI) (1:1000; Sigma-Aldrich). Then, the images were obtained using the Cytation 3 Cell Imaging Multimode reader (BioTek, AHSI, Milan Italy) and analyzed by the Gen5 software (BioTek, AHSI, Milan Italy).

### Phalloidin staining

Cells were exposed to treatments for 16 h, washed twice with PBS, fixed in 4% paraformaldehyde in PBS for 10 min, washed briefly with PBS, then incubated with Phalloidin-Fluorescent Conjugate (Santa Cruz Biotechnology). The images were obtained using the Cytation 3 Cell Imaging Multimode reader (BioTek, AHSI, Milan Italy) and analyzed by the Gen5 software (BioTek, AHSI, Milan Italy).

### Enzyme-linked Immunosorbent assay

The concentrations of IL-1β in supernatants from hypoxia-treated MDA-MB-231 cells were measured using human IL-1β ELISA Kit (Thermo Scientific, Monza Italy), according to the manufacturer’s instructions. The plates were read at 450 nm on a Microplate Spectrophotometer Epoch™ (BioTek, AHSI, Milan Italy).

### DCFDA fluorescence measurement of ROS

The non-fluorescent 2′,7′-dichlorofluorescin diacetate (DCFDA) probe was used to evaluate intracellular ROS production. Briefly, cells were incubated with 10 μM DCFDA (Sigma-Aldrich) at 37 °C for 30 min, washed with PBS, and then exposed to treatments for 15 min, as indicated. Cells were then washed with PBS, and the images were obtained using the Cytation 3 Cell Imaging Multimode reader (BioTek, AHSI, Milan Italy) and analyzed by the Gen5 software (BioTek, AHSI, Milan Italy).

### Conditioned medium

MDA-MB-231 cells were cultured under normal or low oxygen tension (2%) for 16 h. Thereafter, the supernatants were collected, centrifuged at 3500 rpm for 5 min to remove cell debris and used as conditioned medium in the appropriate experiments.

### Cell spreading assay

Cells were treated for 16 h, trypsinized and seeded onto fibronectin (5 μg/ml) coated 96-well plate at a density of 2 × 10^5^ cells/ml and incubated at 37 °C in a humidified incubator containing 5% CO2. Phase-contrast images were captured after 15 min and 60 min. As previously reported [33], round bright cells were considered unspread, whereas spread cells were defined as those cells that had lost their phase-bright appearance and had readily distinguishable nucleus and cytoplasm and quantified by counting six randomly selected fields in each well under a phase-contrast microscope.

### Invasion assay

Transwell 8 μm polycarbonate membrane (Costar, Sigma-Aldrich, Milan, Italy) was used to evaluate in vitro cell invasion. 5 × 10^4^ cells in 300 μL serum-free medium were seeded in the upper chamber, coated with Corning® Matrigel® Growth Factor Reduced (GFR) Basement Membrane Matrix (Biogenerica, Catania, Italy) (diluted with serum-free medium at a ratio of 1:3). For invasion assays in MDA-MB-231 cells, complete medium was added to the bottom chambers in the presence of treatments where required, then cells were cultured under normoxia or hypoxia for 16 h. For invasion assays in CAFs, conditioned medium collected from MDA-MB-231 cells previously exposed to hypoxia for 16 h was added to the bottom chambers in the presence of treatments, where required. Cells on the upper surface of the membrane were then removed by wiping with Q-tip, and invaded cells were fixed with 100% methanol, stained with Giemsa (Sigma-Aldrich, Milan, Italy), photographed using Cytation 3 Cell Imaging Multimode Reader (BioTek, AHSI, Milan Italy) and counted using the WCIF ImageJ software.

### Spheroid formation assay

For spheroid generation, 100 μL/well of MDA-MB 231 cell suspensions (1 × 10^4^) were dispensed into 2% agar-coated 24-well plates. Three days after seeding, tumor spheroids (a single spheroid per well) were exposed to treatments and a 50% medium and treatment replenishment was performed every 2 days. Images were obtained on day 20 using a conventional inverted microscope, thereafter cell number per spheroid was determined by trypsinizing three different spheroids, mixing the cell suspension with trypan blue and counting the number of viable cells. The total number of cells obtained was divided by the number of trypsinized spheroids.

### Statistical analysis

The statistical analysis was performed using ANOVA followed by Newman-Keuls’ test to determine differences in means. The bioinformatics analyses, including t-tests, box plots and scatter plots, were performed using the R tidyverse package (https://www.tidyverse.org/packages/). *p* < 0.05 and *p* < 0.01 were considered statistically significant.

## Results

### Expression levels of HIF-1α and IL-1β are correlated in TNBC

**Fig. 1:**
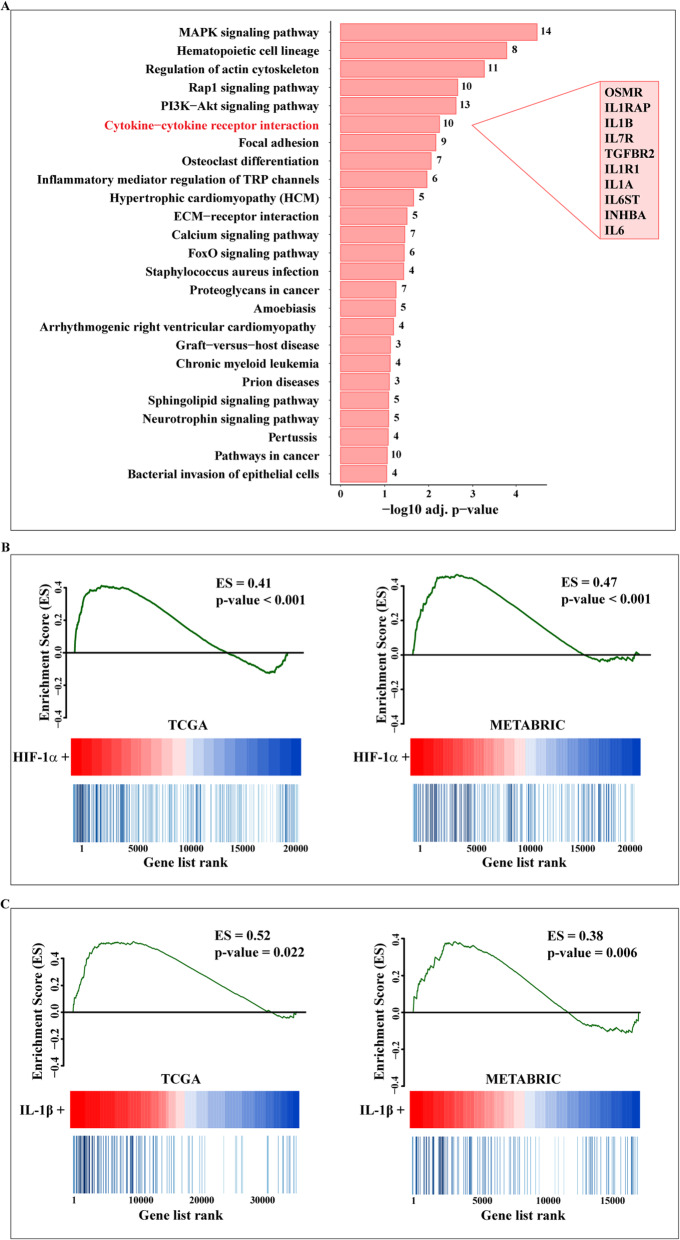
**a** The “Cytokine-cytokine receptor interaction” signaling is a prominent transduction pathway associated with the expression of HIF-1α in TNBC patients. The top 250 genes correlated with HIF-1α in the TCGA dataset were clustered using the KEGG pathway enrichment analysis. The *x*-axis and the *y*-axis indicate respectively the −log10-adjusted *p*-value and the different KEGG pathways. The number of the genes represented in the identified pathways is plotted on the right of each bar. The genes belonging to the “Cytokine-cytokine receptor interaction” pathway (listed in the pink box) were ordered by their HIF-1α correlation coefficients (from high to low) (see Additional file 1). **b** GSEA confirms an enrichment of the genes belonging to the “Cytokine-cytokine receptor interaction” pathway in the TNBC patients with high HIF-1α levels, as indicated. The patients of the TCGA and METABRIC datasets were ranked in accordance with the differential HIF-1α expression levels. **c** GSEA reveals an enrichment of the “HIF-1 signaling” genes in the TNBC patients with high IL-1β expression. Patients of the TCGA and METABRIC datasets were ranked in accordance with the differential IL-1β expression levels. Enrichment scores (ES) and relative *p*-values are plotted

**Fig. 2:**
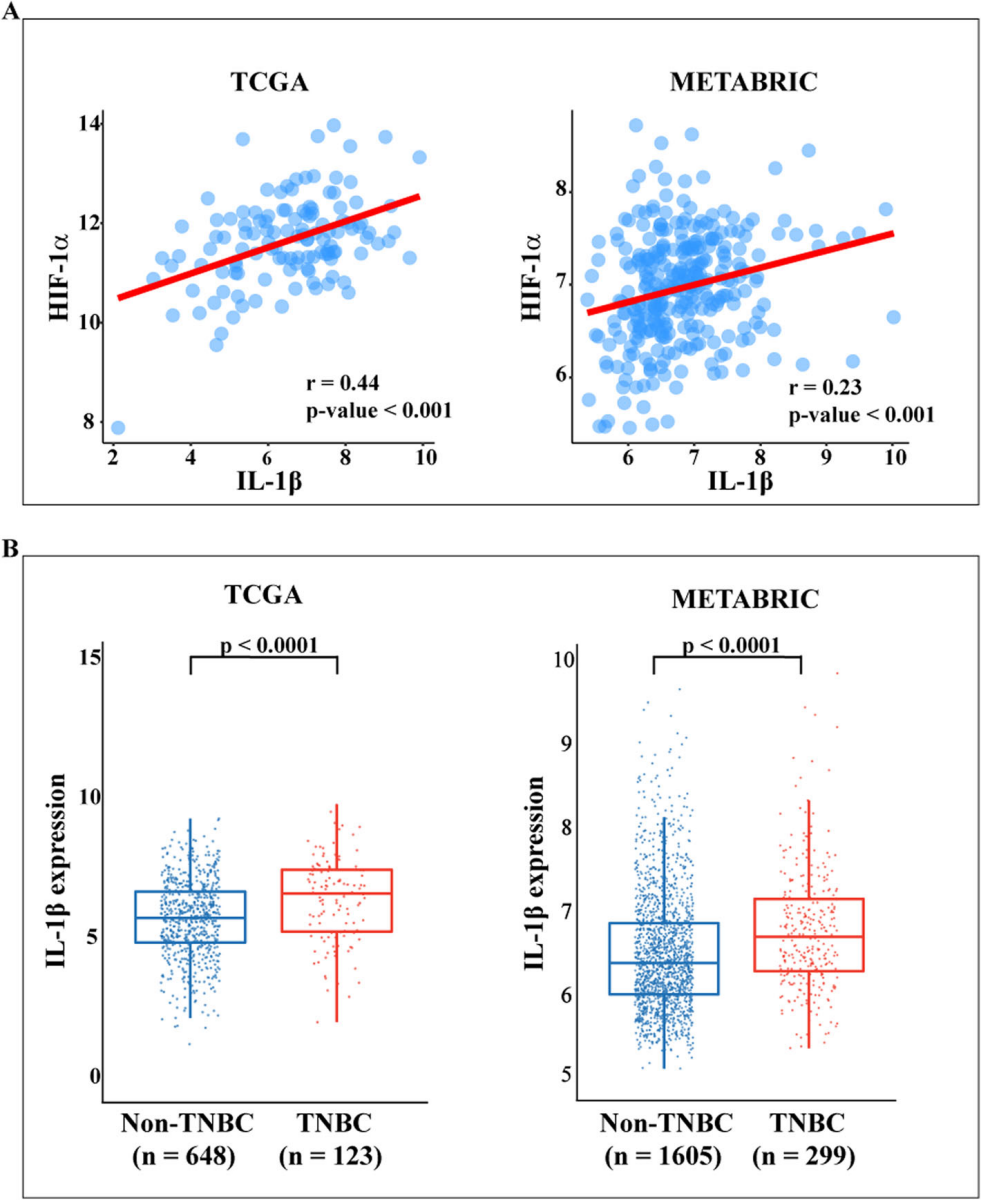
**a** Scatter plots depicting the correlation between HIF-1α expression and IL-1β levels in TNBC patients of the TCGA and METABRIC cohorts. The Pearson correlation coefficients (r) and the relative p-values are shown in each panel. **b** Box plots showing the differential IL-1β expression levels in non-TNBC and TNBC patients, as found in the TCGA and METABRIC datasets. The number of patients and *p*-values are reported in each panel

It is well acknowledged that the transcription factor HIF-1α acts as a molecular sensor within the hypoxic breast tumor microenvironment toward aggressive cancer features [7]. Hence, we began our study evaluating the gene signature associated with HIF-1α in TNBC patients that are characterized by a poor prognosis [34]. Exploring the TCGA cohort of TNBC patients, the 250 genes mostly correlated with HIF-1α were first ranked by Pearson correlation coefficient. In order to investigate the biological significance of these genes, KEGG pathway analysis was then performed using DAVID. The top 250 HIF-1α correlated genes were enriched in a set of pathways, as schematically reported in Fig. 1a. The “Cytokine-cytokine receptor interaction” transduction pathway, including IL1B, IL1A, IL1R, IL6, IL6ST, IL7R, IL1RAP, INHBA, OSMR and TGFBR2 that are strongly associated with breast cancer progression [35–41], was found significantly correlated with HIF-1α. Next, we performed gene set enrichment analysis (GSEA) in order to explore the “Cytokine-cytokine receptor interaction” expression profile according to the high and low HIF-1α phenotypes of both TCGA and METABRIC cohorts of TNBC patients. It is worth noting that the genes included in the “Cytokine-cytokine receptor interaction” pathway were found enriched in the group of patients showing high HIF-1α levels (Fig. 1b). As IL-1β may contribute to breast cancer development and metastasis [37] and may regulate the migratory phenotype of TNBC cells through HIF-1α [42, 43], we sought to evaluate whether IL-1β could be involved in the activation of a hypoxia-related gene signature in TNBC patients. In this regard, we ascertained that the genes included in the “HIF-1 signaling pathway” of the KEGG database are significantly enriched in the TNBC cohorts displaying high IL-1β expression of both TCGA and METABRIC datasets (Fig. 1c). Further corroborating these observations, a positive correlation between IL-1β and HIF-1α levels was found in the TNBC cohorts of both databases (Fig. 2a). Exploring the clinical significance of IL-1β in TNBC patients, we assessed that the IL-1β expression levels are significantly higher in TNBC compared to non-TNBC in both TCGA and METABRIC datasets (Fig. 2b). Overall, the aforementioned data suggests that IL-1β is engaged by HIF-1α toward the development of the aggressive TNBC.

### GPER is involved in the up-regulation of IL-1β mediated by HIF-1α upon hypoxia

**Fig. 3:**
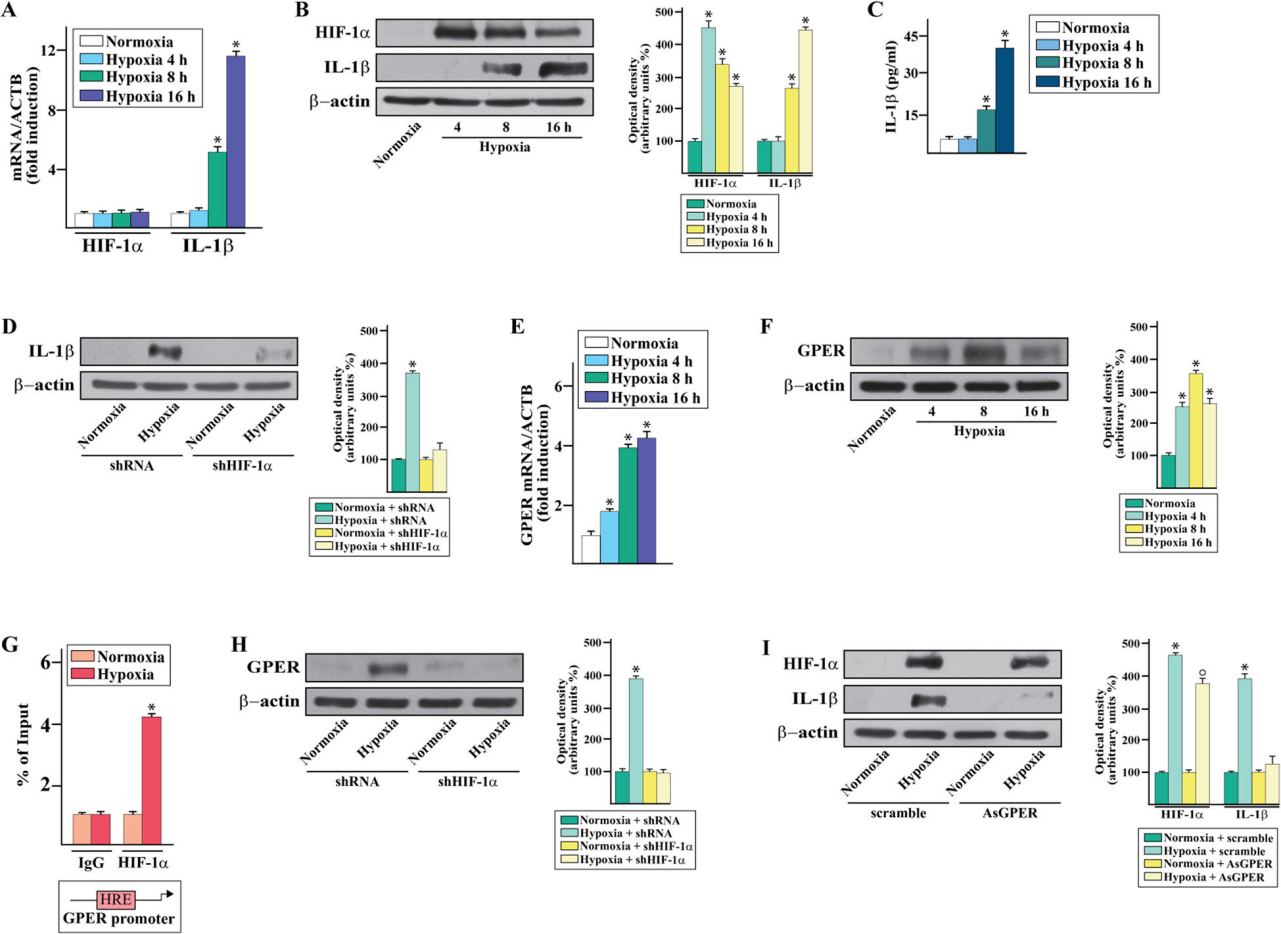
The HIF-1α/GPER signaling is involved in the expression of IL-1β induced by hypoxia. mRNA (**a**) and protein (**b**) expression of HIF-1α and IL-1β evaluated respectively by real-time PCR and immunoblotting in MDA-MB-231 breast cancer cells cultured upon normoxia or hypoxia (2% O2). In RNA experiments, values are normalized to the actin beta (ACTB) expression and shown as fold changes of mRNA expression induced by hypoxia compared to normoxic cells. **c** IL-1β levels evaluated by ELISA in the supernatants collected from MDA-MB-231 cells cultured upon normoxia or hypoxia. **d** The up-regulation of IL-1β observed in MDA-MB-231 cells cultured upon hypoxia is no longer evident silencing HIF-1α. mRNA (**e**) and protein (**f**) expression of GPER evaluated in MDA-MB-231 cells cultured upon normoxia or hypoxia, as evaluated by real-time PCR and immunoblotting, respectively. **g** Recruitment of HIF-1α to the HRE site located within the GPER promoter sequence in MDA-MB-231 cells cultured upon hypoxia. In control samples nonspecific IgGs were used instead of the primary antibody. The amplified sequences were evaluated by real-time PCR. **h** The up-regulation of GPER observed in MDA-MB-231 cells exposed to hypoxia was abrogated silencing HIF-1α. **i** Immunoblots of HIF-1α and IL-1β from GPER-silenced MDA-MB-231 cells exposed to hypoxia. Side panels show densitometric analysis of the blots normalized to β-actin. Values represent the mean ± SD of three independent experiments performed in triplicate. (*) and (○) indicate *p* < 0.05 for cells cultured under normoxia versus cells cultured under hypoxia

**Fig. 4:**
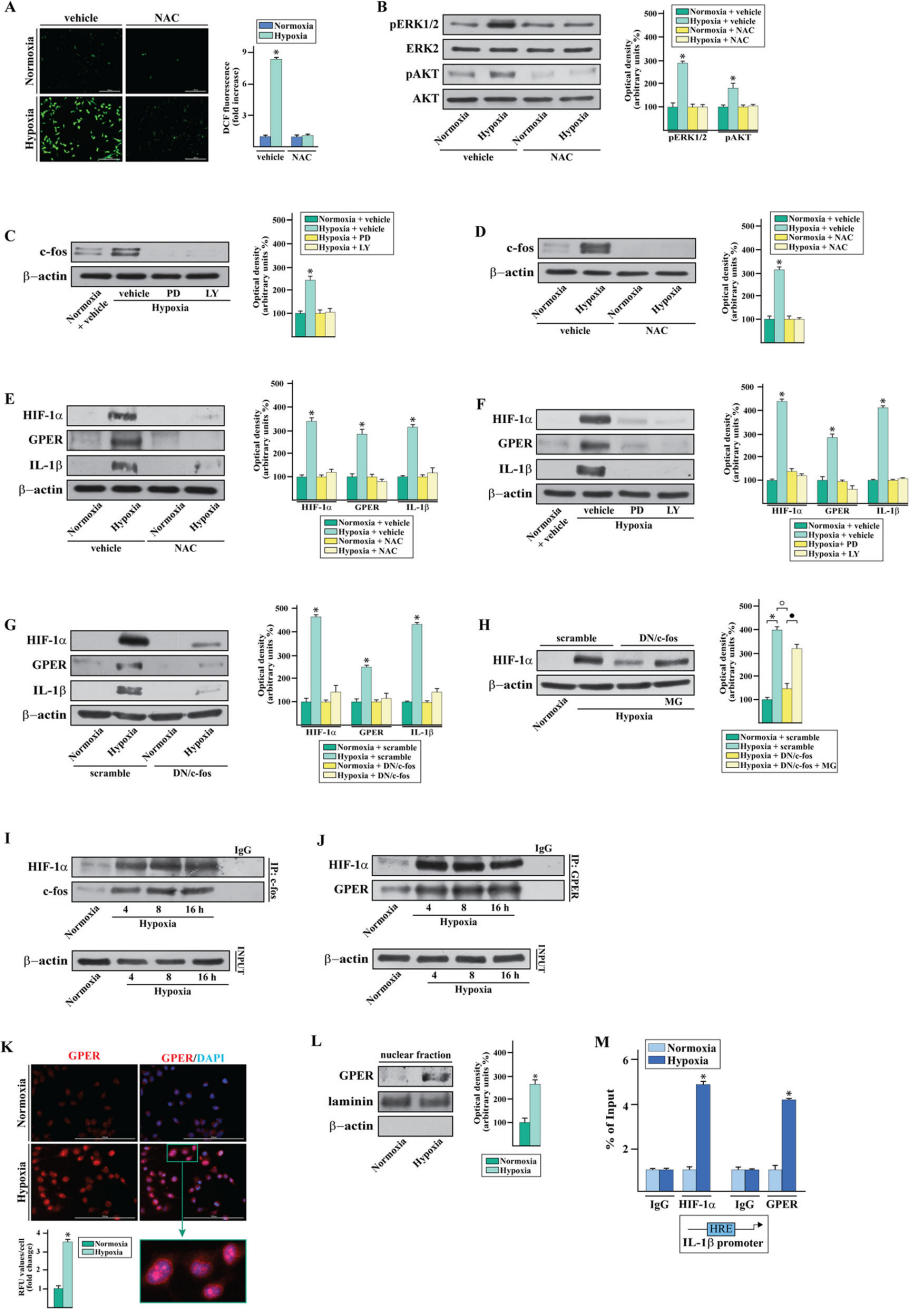
The ERK1/2 and AKT transduction pathways along with c-fos, HIF-1α and GPER are involved in the up-regulation of IL-1β induced by hypoxia. **a** ROS generation in MDA-MB-231 cells cultured upon normoxia or 15 min hypoxia (2% O2) in the presence or absence of the free radical scavenger NAC, as evaluated using the fluorescent probe DCF-DA. Scale bar 200 μM. Side panel shows the quantitative measurement of intracellular ROS (DCF fluorescence obtained in normoxic cells was set as one-fold induction upon which ROS levels induced by hypoxia was calculated). Values represent the mean ± SD of three independent experiments performed in triplicate. **b** The ERK1/2 and AKT activation induced by 15 min hypoxia in MDA-MB-231 cells is no longer evident in the presence of NAC. Side panels show densitometric analysis of the blots normalized to ERK2 and AKT that served as loading control, as indicated. **c** Immunoblot of c-fos from MDA-MB-231 cells cultured upon normoxia or hypoxia and in the presence of MEK inhibitor PD98059 (PD) or PI3K inhibitor LY294,002 (LY). Immunoblots of c-fos (**d**), HIF-1α, GPER and IL-1β (**e**) in MDA-MB-231 cells upon normoxia or hypoxia in the presence or absence of NAC. **f** Immunoblots of HIF-1α, GPER and IL-1β in MDA-MB-231 cells upon normoxia or hypoxia and in the presence of PD or LY. **g** Immunoblots of HIF-1α, GPER and IL-1β in MDA-MB-231 cells transfected with a scramble or a dominant-negative c-fos construct (DN/c-fos) and thereafter exposed to normoxia or hypoxia. **h** The 26S proteasome inhibitor MG132 rescued the HIF-1α repression observed in MDA-MB-231 cells transfected for 24 h with the DN/c-fos construct and exposed to low oxygen tension (2%). Side panels show densitometric analysis of the blots normalized to β-actin. Values represent the mean ± SD of three independent experiments performed in triplicate. **i**-**j** Co-immunoprecipitation assays performed in MDA-MB-231 cells cultured in normoxia or hypoxia, as indicated. Cell lysates were immunoprecipitated with anti-c-fos (**i**) or anti-GPER (**j**) antibodies. Immunocomplexes were analyzed by immunoblot with antibodies against the indicated proteins. In control samples, nonspecific IgG was used instead of the primary antibody. An equal amount of the total lysates (input) was blotted for β-actin as loading control. **k** GPER expression evaluated by immunofluorescence assays in MDA-MB-231 cells cultured upon normoxia or hypoxia. Nuclei were stained by DAPI (blue signal). Fluorescence intensities were quantified in 20 random fields for each condition and results are expressed as fold change of relative fluorescence units (RFU) over cells cultured upon normoxia (set as one-fold induction). Enlarged details are shown in the separate box. **l** Immunoblots of nuclear fraction lysates derived from MDA-MB-231 cells cultured upon normoxia or hypoxia. Side panel shows densitometric analysis of the blots normalized to laminin, which served as a nuclear marker. β-actin served as a cytoplasmic marker. **m** Recruitment of HIF-1α and GPER to the HRE site located within the IL-1β promoter sequence in MDA-MB-231 cells exposed to hypoxia. In control samples nonspecific IgG was used instead of the primary antibody. The amplified sequences were evaluated by real-time PCR. Values represent the mean ± SD of three independent experiments performed in triplicate. (*), (○) *p* < 0.05 for cells cultured upon normoxia versus cells cultured upon hypoxia

On the basis of the aforementioned findings and in order to provide novel insights on the regulation of IL-1β by hypoxia in TNBC, we ascertained that a low oxygen tension (2% O2, thereafter mentioned as hypoxia) induces the mRNA (Fig. 3a) and protein (Fig. 3b) expression of IL-1β in MDA-MB-231 cells, which are a well-recognized model system of TNBC. Upon hypoxic conditions, the mRNA levels of HIF-1α did not display any change (Fig. 3a) whereas an increased protein expression was observed (Fig. 3b), in accordance with the known ability of hypoxia to decrease the HIF-1α ubiquitination and degradation [9]. Next, performing ELISA assays we determined that the secretion of IL-1β increases in the medium of MDA-MB-231 cells cultured under hypoxia (Fig. 3c). Remarkably, the silencing of HIF-1α abolished the expression of IL-1β prompted by hypoxia, suggesting that HIF-1α is involved in the up-regulation of IL-1β in MDA-MB-231 cells exposed to hypoxic conditions. In accordance with previous findings showing that HIF-1α can be induced by proinflammatory stimuli including IL-1β [43], we ascertained that IL-1β boosts the accumulation of HIF-1α in MDA-MB-231 cells (Additional file 2). Worthy, these results suggest that the HIF-1α-dependent induction of IL-1β upon hypoxic conditions may generate a feed-forward loop that further contributes to the stimulatory interaction between HIF-1α and IL-1β signaling. Reminiscing our previous data on the action of GPER in hypoxic conditions [13, 14], we assessed that hypoxia triggers the up-regulation of GPER mRNA (Fig. 3e) and protein (Fig. 3f) levels in a time-dependent manner in MDA-MB-231 cells. By chromatin immunoprecipitation (ChIP) assay, we then found that HIF-1α is recruited to the GPER promoter region in MDA-MB-231 cells exposed to hypoxia (Fig. 3g). Moreover, the silencing of HIF-1α prevented the protein induction of GPER upon a 16 h exposure to hypoxic conditions (Fig. 3h), indicating that HIF-1α is required for the transcription of GPER in MDA-MB-231 cells exposed to hypoxia. Next, the hypoxia increased HIF-1α levels were not altered by the silencing of GPER that instead abolished the induction of IL-1β (Fig. 3i). Together, these findings suggest that upon hypoxia HIF-1α is involved in the up-regulation of GPER, which then cooperates with HIF-1α toward the regulation of IL-1β. In order to evaluate the transduction mechanism mediating the aforementioned responses, we first assessed that a short exposure of MDA-MB-231 cells to hypoxia does increase the levels of reactive oxygen species (ROS) (Fig. 4a), which are required for the stabilization of HIF-1α and the subsequent transduction of the hypoxia-mediated signaling [44]. In the aforementioned experimental condition, we next ascertained that the activation of two main transduction regulators of HIF-1α, namely ERK1/2 and Akt [45, 46], is prevented in the presence of the ROS scavenger NAC (Fig. 4b). As c-fos expression is a suitable molecular sensor of both GPER and HIF-1α transduction signaling [14, 47, 48], we also determined that the induction of c-fos protein levels observed upon a 4 h exposure to hypoxia is no longer evident in the presence of the MEK inhibitor PD, the PI3K inhibitor LY (Fig. 4c) and NAC (Fig. 4d). Likewise, the increase of HIF-1α, GPER and IL-1β protein levels upon a 16 h exposure to hypoxia using either NAC (Fig. 4e) or PD and LY (Fig. 4f), suggesting that the ROS activity triggers the ERK1/2/Akt/c-fos transduction signaling toward the hypoxic regulation of HIF-1α, GPER and IL-1β expression. In accordance with these findings, the transfection in MDA-MB-231 cells of a plasmid encoding a mutant of c-fos, namely dominant negative c-fos (DN/c-fos), abrogated the up-regulation of HIF-1α, GPER and IL-1β triggered by a 16 h exposure to hypoxia (Fig. 4g). In order to evaluate whether the reduction of HIF-1α protein levels triggered in the aforementioned experimental condition by the DN/c-fos construct is associated with the HIF-1α proteasome-dependent degradation, the MDA-MB-231 cells were treated for 16 h with the proteasome inhibitor MG132. Notably, the MG132 rescued the effects of the DN/c-fos construct on the HIF-1α protein levels (Fig. 4h), suggesting that c-fos is involved in the complex HIF-1α biological regulation at the proteasomal level. By co-immunoprecipitation studies, we also found that hypoxia stimulates a direct interaction between c-fos and HIF-1α in MDA-MB-231 cells (Fig. 4i), indicating that the action of c-fos on HIF-1α stabilization at least in part occurs through a physical interaction between these two factors. Considering that in our previous study the functional cooperation between HIF-1α and GPER triggered the expression of certain genes in hypoxic conditions [13], we first ascertained that HIF-1α co-immunoprecipitates with GPER in MDA-MB-231 cells exposed to hypoxia (Fig. 4j). Thereafter, we evidenced the accumulation of GPER in the nuclear compartment of MDA-MB-231 cells exposed to hypoxia, as evidenced by both immunofluorescence (Fig. 4k) and subcellular fractionation studies (Fig. 4l). Intriguingly, by ChIP assays we then demonstrated that the exposure to hypoxia induces the recruitment of both HIF-1α and GPER to a HRE site located within the IL-1β promoter sequence (Fig. 4m). Overall, these data suggest that a functional interplay between HIF-1α and GPER may be stimulated by hypoxia toward the transcription of IL-1β in MDA-MB-231 cells.

### Hypoxia triggers a functional cooperation among HIF-1α, GPER and IL-1β/IL1R1 signaling toward a metastatic gene expression profile and invasive properties of TNBC cells

**Fig. 5:**
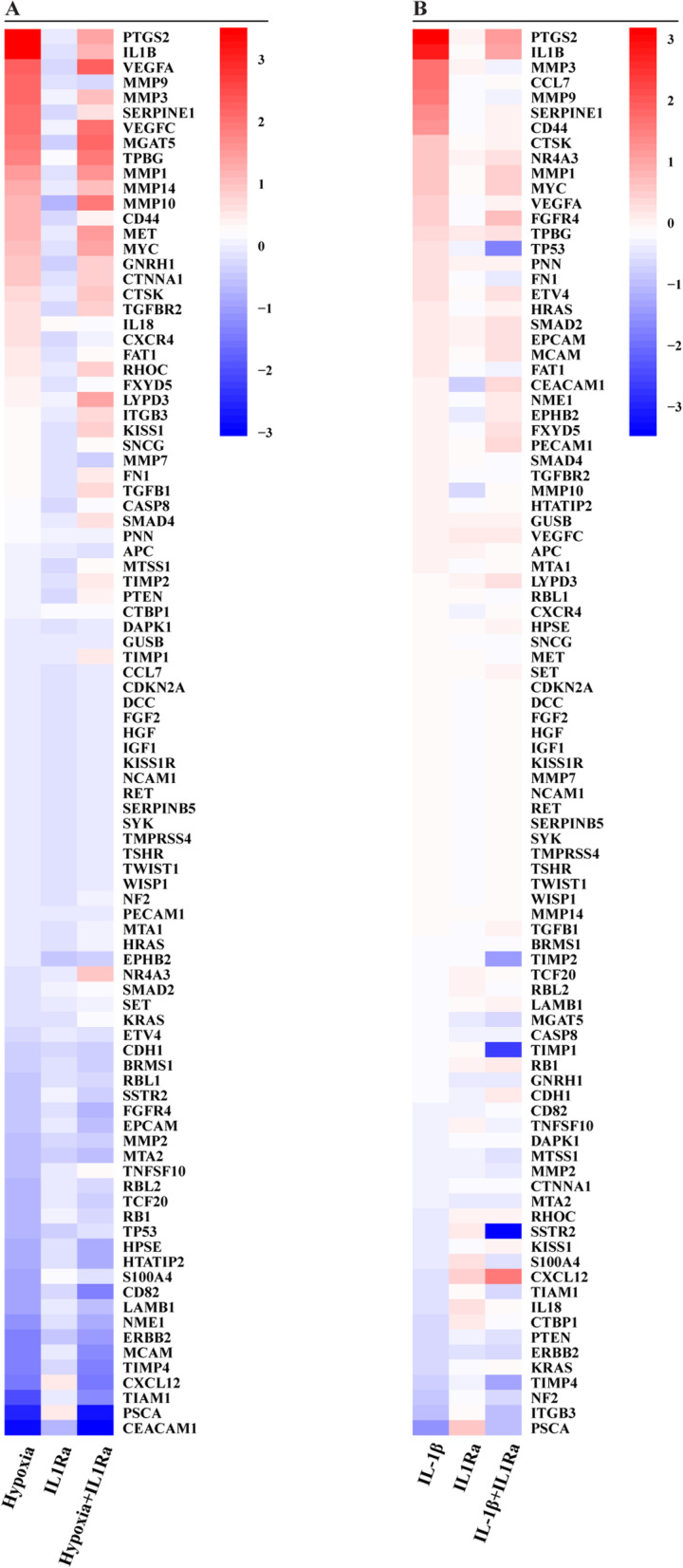
Consistent expression changes of metastasis-related genes are induced by hypoxia and IL-1β in MDA-MB-231 cells, as evaluated by TaqMan™ Human Tumor Metastasis Array. **a** MDA-MB-231 cells were cultured upon normoxia or hypoxia (2% O2) in the presence or absence of the IL1R1 antagonist IL1R1a. **b** MDA-MB-231 cells were treated with vehicle or IL-1β alone or in combination with IL1R1a. Values were normalized to the Glucuronidase Beta (GUSB) expression, the colors indicate the log2 fold changes of gene expression upon the indicated conditions

**Fig. 6:**
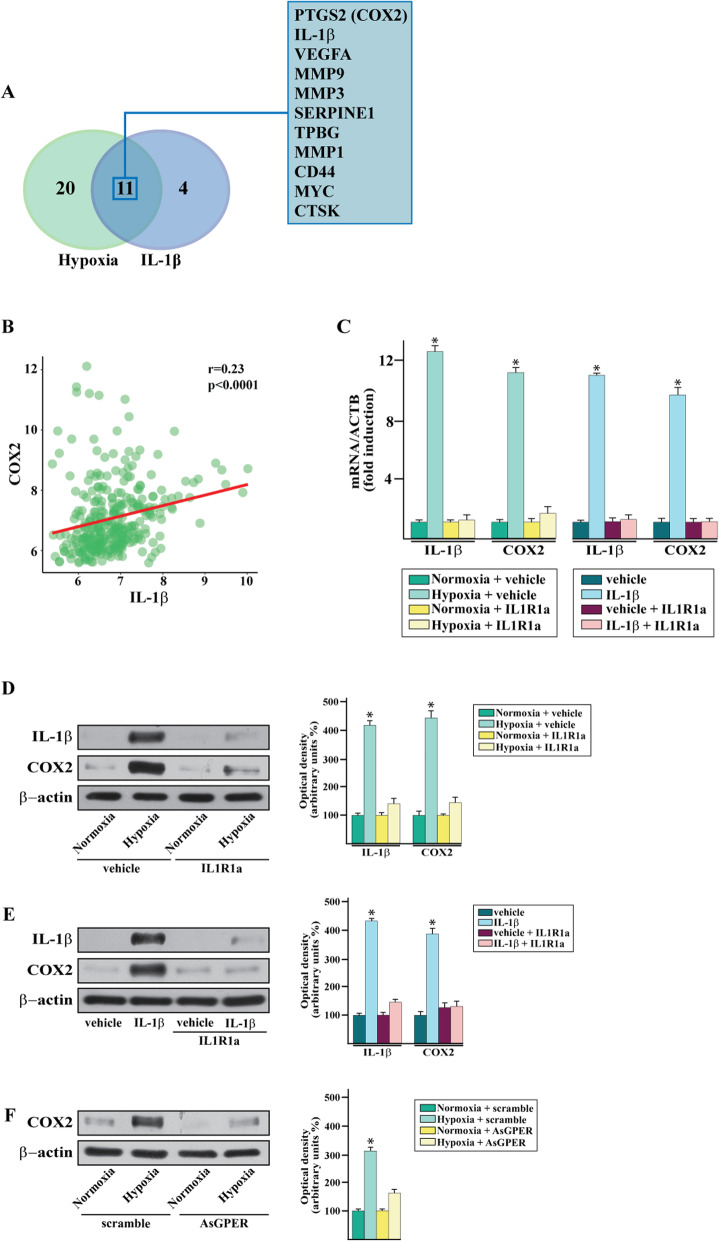
**a** Venn diagram of metastatic genes up-regulated in MDA-MB-231 cells in an exclusive and shared manner upon hypoxia (2% O2) and IL-1β. The genes up-regulated by both hypoxia and IL-1β are listed in the box according to the gene expression changes induced by hypoxia (from high to low) (see Additional file 3). **b** mRNA expression of IL-1β and COX2 in MDA-MB-231 cells cultured upon normoxia and hypoxia or vehicle and IL-1β in the presence or absence of the IL1R1 antagonist IL1R1a, as evaluated by real-time PCR. Values are normalized to the actin beta (ACTB) expression and shown as fold changes of the mRNA expression induced by hypoxia respect to normoxic cells. **c** Scatter plot depicting the correlation between the expression of COX2 and IL-1β in TNBC patients of the METABRIC cohort of patients. The Pearson correlation coefficient (r) and the relative *p*-value are indicated. **d** Immunoblots of IL-1β and COX2 in MDA-MB-231 cells cultured upon normoxia or hypoxia in combination with IL1R1a, as indicated. **e** Immunoblots of IL-1β and COX2 in MDA-MB-231 cells treated with vehicle or IL-1β and in combination with IL1R1a. **f** Immunoblots of COX2 in MDA-MB-231 cells upon normoxia and hypoxia silencing GPER. Side panels show densitometric analysis of the blots normalized to β-actin. Values represent the mean ± SD of three independent experiments performed in triplicate. (*) *p* < 0.05 for cells cultured under normoxia versus cells cultured under hypoxia or cells treated with IL-1β versus vehicle-treated cells

In order to provide further insights into the metastatic gene expression profile triggered by hypoxia through the IL-1β/IL1R1 axis, we performed a TaqMan Gene Expression Assay, which consists of a Human Tumor Metastasis Array. In this vein, MDA-MB-231 cells were exposed for 16 h either to hypoxic conditions (Fig. 5a) or the recombinant human IL-1β (Fig. 5b), in the presence or absence of the IL1R1 antagonist IL1R1a. Considering the genes resulting with at least a 0.25 log2 fold change upon either hypoxia respect to normoxia-exposed cells (Fig. 5a) or IL-1β respect to vehicle-treated cells (Fig. 5b), 31 and 15 metastatic genes induced respectively by hypoxia and IL-1β were identified (Fig. 6a). Among the 11 genes up-regulated by either hypoxia or IL-1β as depicted in the Venn diagram (Fig. 6a and Additional file 3), we then focused on the regulation of IL-1β and its target gene cyclooxygenase 2 (COX2) considering that their strong increase was abrogated using the IL1R1 antagonist IL1R1a (Fig.5a-b). First, we ascertained that a positive correlation does exist between IL-1β and COX2 levels in TNBC patients of the METABRIC dataset (Fig. 6b). Thereafter, we confirmed by real-time and western blotting assays that the IL1R1 antagonist IL1R1a prevents the mRNA (Fig. 6c) and protein (Fig. 6d-e) expression of both IL-1β and COX2 induced by a 16 h exposure to either hypoxia or IL-1β treatment, in accordance with previous evidence demonstrating that IL-1β may auto-regulate its own production [49]. Furthermore, we demonstrated that the COX2 protein induction by a 16 h hypoxic condition is prevented by silencing GPER (Fig. 6f), indicating that GPER is involved not only in the above described IL-1β expression but also in the increase of COX2 upon hypoxia.

**Fig. 7:**
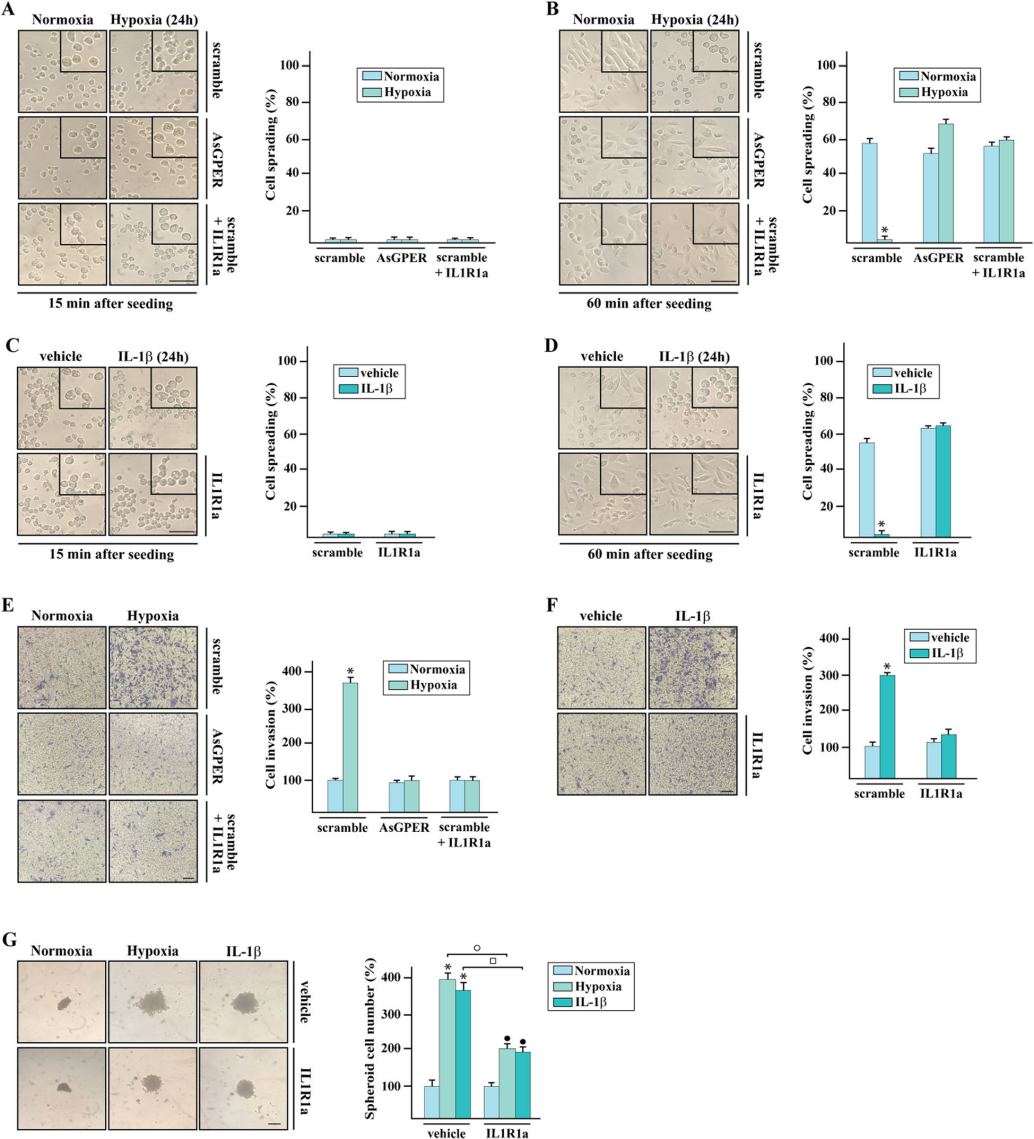
The IL-1β/IL1R1 system is involved in the invasive and proliferative features induced by hypoxia in MDA-MB-231 cells. **a**-**b** Cell spreading was evaluated in MDA-MB-231 cells transfected with scramble or AsGPER and exposed to normoxia or hypoxia (2% O2) in the presence or absence of IL1R1a. Cells were then trypsinized, plated onto coverslips coated with fibronectin and the morphology was recorded after 15 min (**a**) or 60 min (**b**). **c**-**d** Cell spreading was evaluated in MDA-MB-231 cells treated with vehicle and IL-1β alone or in combination with IL1R1a, as indicated. Cells were then trypsinized, plated onto coverslips coated with fibronectin and the morphology was recorded after 15 min (**c**) or 60 min (**d**). Scale bar 50 μM. Side panels show quantification of cell spreading, as indicated. Data are representative of three independent experiments performed in triplicate. **e**-**f** Transwell Matrigel invasion assay in MDA-MB-231 cells transfected with scramble or AsGPER and then cultured in normoxia or hypoxia (**e**) or treated with vehicle or IL-1β (**f**) alone or in combination with IL1R1a, as indicated. Cells were counted in at least 10 random fields in three independent experiments performed in triplicate, as quantified in the side panels. Scale bar 200 μM. **g** Spheroid formation assay in MDA-MB-231 cells exposed to hypoxia and IL-1β alone or in combination with IL1R1a. Scale bar 100 μm. Side panel shows the quantification of cell growth. Values represent the mean ± SD of three independent experiments performed in triplicate. (*), (○), (□) *p* < 0.05 for cells cultured under normoxia versus cells cultured under hypoxia or treated with IL-1β versus vehicle-treated cells

A hypoxic environment may lower cell-ECM adhesion prompting cell motility and the epithelial to mesenchymal transition toward aggressive breast cancer phenotypes [50, 51]. Hence, we evaluated the contribution of GPER and IL-1β/IL1R1 mediated signaling in the metastasis-related responses induced by the hypoxia-stimulated IL-1β action. In this regard, we assessed that MDA-MB-231 cells cultured under hypoxic conditions exhibit a reduced spreading ability on fibronectin 60 min after seeding respect to cells grown under normoxic conditions (Fig. 7a-b). Notably, this effect was no longer evident silencing GPER as well as using the IL1R1 antagonist IL1R1a (Fig. 7a-b). Results similar to those mentioned above were observed treating cells with IL-1β (Fig. 7c-d). Together, these findings suggest that both GPER and IL-1β/IL1R axis contribute to hypoxia-decreased cancer cell spreading on the ECM protein fibronectin, according to previous data correlating the hypoxia-lowered cell-ECM adhesion to an increased invasion, intravasation and metastasis [52, 53]. Next, the invasive effects prompted by both hypoxia and IL-1β treatment were prevented not only by silencing GPER but also using the IL1R1 antagonist IL1R1a, as evaluated by Transwell Matrigel invasion assays (Fig. 7e-f). Moreover, both GPER silencing and IL1R1a lessened the spheroid expansion upon hypoxia and IL-1β treatment (Fig. 7g). Overall, these findings may suggest that the signaling network of both HIF-1α/GPER and IL-1β/IL1R, may lead to an auto-regulatory loop of IL-1β toward invasive and metastatic properties of hypoxic TNBC cells.

### The hypoxic regulation of the IL-1β/IL1R1 signaling in TNBC cells promotes an invasive CAFs phenotype

**Fig. 8:**
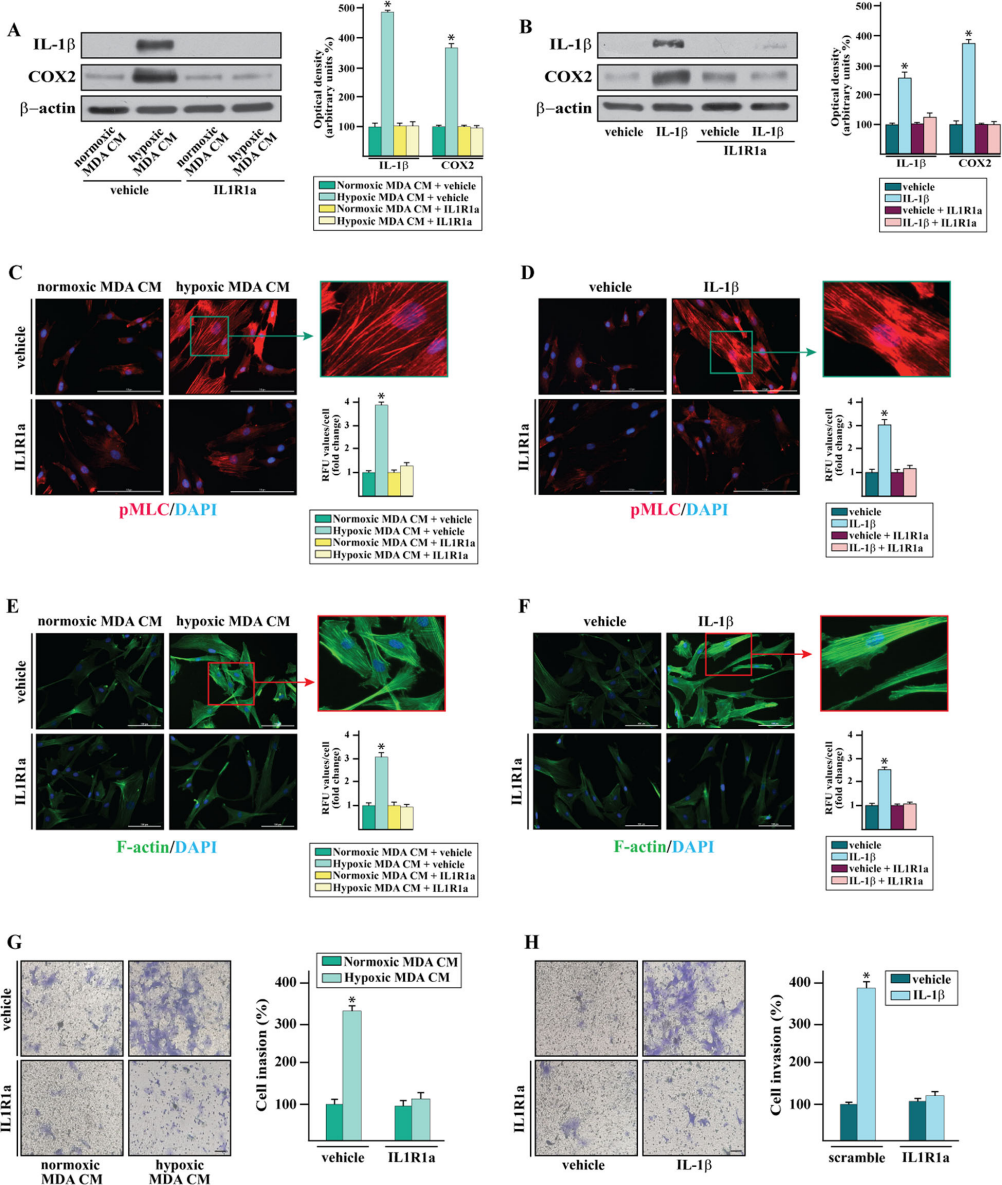
The paracrine action of the IL-1β/IL1R1 axis promotes the actin-myosin contractility and the invasion of CAFs. **a**-**b** Immunoblots of IL-1β and COX2 in CAFs exposed to conditioned medium (CM) collected from MDA-MB-231 cells cultured upon normoxia or hypoxia (2% O2) (**a**) and in CAFs treated with vehicle or IL-1β (**b**), in the presence or absence of IL1R1a. **c**-**d** Phosphorylation of MLC in CAFs cultured with conditioned medium (CM) collected from MDA-MB-231 cells exposed to normoxia and hypoxia (**c**) or IL-1β (**d**), in the presence or absence of the IL1R1 antagonist IL1R1a. Nuclei were stained by DAPI (blue signal). **e**-**f** CAFs exposed to conditioned medium (CM) from MDA-MB-231 cells grown upon normoxia and hypoxia (**e**) or treated with IL-1β (**f**), in the presence or absence of the IL1R1 antagonist IL1R1a were stained with FITC-phalloidin to detect F-actin stress fibers (green) and with DAPI to detect nuclei (blue). Fluorescence intensities of pMLC and the number of stress fibers/cell was quantified based on F-actin staining in 20 random fields for each condition; results are expressed as fold change of relative fluorescence units (RFU). Enlarged details are shown in the separate boxes. Scale bar 100 μM. **g**-**h** Transwell Matrigel invasion assay in CAFs exposed to conditioned medium (CM) collected from MDA-MB-231 cells grown upon normoxia or hypoxia (**g**) or treated with IL-1β (**h**), in the presence or absence of IL1R1a. Cells were counted in at least 10 random fields in three independent experiments performed in triplicate, as quantified in side panels. Scale bar 200 μM. Values represent the mean ± SD of three independent experiments performed in triplicate. (*) *p* < 0.05

On the basis of the aforementioned findings, we sought to provide mechanistic insights into the paracrine IL-1β feedback on important components of the tumor microenvironment as CAFs. In this vein, CAFs were cultured with the conditioned medium (CM) collected from MDA-MB-231 cells, which were exposed to hypoxia. Interestingly, the up-regulation of both IL-1β and COX2 protein levels occurring in CAFs upon this experimental condition (Fig. 8a) was prevented either using the IL1R1 antagonist IL1R1a (Fig. 8a) or the neutralizing IL-1β antibody (Additional file 4). Findings similar to those above mentioned were observed in CAFs treated with IL1β (Fig. 8b). Considering that hypoxia may stimulate breast cancer cells toward invasive features as cell motility, formation of stress fibers and matrix contraction [54], the CM from MDA-MB-231 cells exposed to hypoxia promoted in CAFs the phosphorylation of myosin light chain (MLC) on serine-19 (pMLC^S19^) (Fig. 8c), which is known to be required for the coordination of the actin-myosin contractility [55]. In accordance with these results, an increased MLC phosphorylation was evidenced in CAFs treated with IL1β (Fig. 8d). Moreover, immunofluorescent staining of polymerized actin (F-actin) using FITC-conjugated phalloidin revealed an augmented formation of stress fibers upon exposure of CAFs to the CM from hypoxia-stimulated MDA-MB-231 cells (Fig. 8e) as well as upon treatment with IL-1β (Fig. 8d). Notable, both pMLC^S19^ phosphorylation and the increased formation of actin stress fibers triggered in CAFs by the CM from hypoxic MDA-MB-231 cells (Fig. 8c, e) and IL-1β treatment (Fig. 8d, f), were abolished in the presence of the IL1R1 antagonist IL1R1a. Then, we observed that the invasion of CAFs promoted by the CM from hypoxia-exposed MDA-MB-231 cells (Fig. 8g) or by IL-1β treatment (Fig. 8h), was no longer evident in the presence of IL1R1a. Altogether, these results show that the IL-1β production by the hypoxic TNBC cells may trigger a paracrine feed-forward signaling that stimulates malignant features in main components of the breast tumor microenvironment as CAFs.

## Discussion

In the current study we have provided novel insights regarding the hypoxia-mediated regulation of the IL-1β/IL1R1 transduction signaling in TNBC cells and patient-derived breast CAFs. First, we assessed that the genes belonging to the “Cytokine-cytokine receptor interaction” pathway are both associated with HIF-1α expression in human TNBC samples and enriched in patients showing high HIF-1α levels. Thereafter, we found that the genes included in the “HIF-1 signaling pathway” are enriched in the TNBC cohort displaying high levels of IL-1β, which is a main component of the “Cytokine-cytokine receptor interaction” pathway. Next, we ascertained that IL-1β expression is higher in TNBC respect to non-TNBC samples and correlates with HIF-1α in TNBC patients. Mechanistically, we then assessed that the HIF-1α/GPER transduction signaling is involved in the hypoxia-regulated action of IL-1β, which triggers both a metastatic gene signature and invasive features in TNBC cells. Focusing on the functional interaction that occurs between tumor cells and the surrounding stroma, we also determined that IL-1β secreted by hypoxic TNBC cells may promote a feed-forward loop engaging its cognate receptor IL1R1 in breast CAFs. Corroborating these findings, we determined that the conditioned medium collected from TNBC cells exposed to hypoxia stimulates in CAFs the actin polymerization and the MLC phosphorylation, which characterize the attainment of invasive phenotypes.

Intratumoral hypoxia, which is a common feature in solid cancers as breast cancer, is associated with an increased risk of metastatic aggressiveness and patient mortality [7]. Of note, cancer cells can adapt to low oxygen tension activating the hypoxia-inducible factors that mainly drive a peculiar gene expression signature [8]. As well-known, the master regulators of the cellular adaptation to hypoxia consist of the oxygen-regulated HIF-1α, HIF-2α and HIF-3α subunits and the constitutively expressed HIF-1β subunit [8]. The HIF-1α, 2α and 3α subunits are subjected to oxygen-dependent processes like prolyl hydroxylation, ubiquitination and proteasomal degradation, which are inhibited upon hypoxia that triggers the post-translational stabilization and the HIF-α protein accumulation [9]. Subsequently, HIF-1α binds to the hypoxia response elements regulating the transcription of genes implicated in critical aspects of cancer biology as angiogenesis, stem cell maintenance, metabolic reprogramming, epithelial-mesenchymal transition, invasion, metastasis and resistance to therapy [9, 56, 57]. Likewise, hypoxia-activated HIF-1α plays a role in the tumor-related inflammation leading to worse clinical outcomes in diverse types of cancers including breast tumors [58]. These events may occur through the HIF-1α dependent regulation of signaling mediators and inflammatory genes in both cancer and neighboring cells within the tumor microenvironment [58]. Moreover, the pro-tumorigenic action of HIF-1α may occur through its interaction with various signaling pathways like transforming growth factor-β, Wnt/β-catenin, Notch and GPCRs [12, 59–61]. In this regard, GPER was shown to be involved together with HIF-1α in the cell adaptation to low oxygen tension in diverse tumors as breast cancer [13, 14]. In particular, hypoxia-activated HIF-1α was found to cooperate with GPER in breast cancer cells and CAFs toward gene expression changes and relevant biological responses [13]. Intriguingly, GPER was also shown to trigger the activation of the IL-1β/IL1R1 transduction signaling, which in turn promoted inflammatory responses and aggressive features of breast cancer cells [26]. Further corroborating these findings, we here demonstrate that a functional liaison between HIF-α and GPER mediates the induction of IL-1β in TNBC cells exposed to hypoxic conditions. In particular, we first evidenced that the up-regulation of GPER upon hypoxia occurs through the recruitment of HIF-1α to a HRE site located within the GPER promoter sequence in TNBC cells. Then, we assessed that in hypoxic TNBC cells HIF-1α and GPER are both recruited to the HRE site located within the IL-1β promoter sequence. It is worth mentioning that many studies have demonstrated that GPER is detectable not only at the plasma membrane level, but also in the intracellular and nuclear compartments [13, 62–66]. As it concerns the signaling cascade triggering the aforementioned findings, we demonstrated that the ROS-dependent ERK1/2 and AKT activation together with the ensuing c-fos induction contribute to the stabilization of HIF-1α as well as the up-regulation of GPER and IL-1β in hypoxic TNBC cells. Co-immunoprecipitation assays also revealed that c-fos both interacts directly and protects HIF-1α from the proteasomal degradation.

As largely reported, TNBC patients show an elevated incidence of metastatic processes and low survival rates [67, 68]. Unfortunately, TNBCs remain to be fully characterized toward the assessment of reliable prognostic markers and drug targets [69, 70]. Of note, “omics” technologies have recently provided intriguing insights into the molecular landscape and the heterogeneous clinical features of TNBC, leading to the identification of potentially actionable targets [71, 72]. In this scenario, the HIF-dependent signaling has been reported to be particularly active in TNBCs, suggesting that hypoxia may play a relevant role toward the peculiar features of TNBCs [5, 6]. Likewise, it has been ascertained that the tumor-stroma inflammation network may prompt the pro-metastatic phenotypes in TNBCs [73]. In this regard, it should be mentioned that IL-1β acts as an important effector of the oncogene-driven pathways linking inflammation to cancer [74, 75]. Indeed, IL-1β is considered as a master cytokine in the progression of breast cancer as its production has been found correlated with advanced diseases, whereas the IL1R1 inhibition by the antagonist anakinra was shown to prevent the growth of breast cancer and the bone metastasis in mouse models [25, 37, 42, 76, 77]. Further corroborating these data, IL-1β was found to contribute to the hypoxia-mediated angiogenesis through the up-regulation of HIF-1α [78]. In the framework of these findings, we have here provided novel evidence regarding the hypoxic-induced IL-1β expression and function toward the metastasis-related gene profile as well as the growth and invasive properties of TNBC cells.

COX2 is the key enzyme in eicosanoid biosynthesis and the master switch in the activation of the inflammatory responses [79]. The induction of COX2 by inflammatory stimuli, including cytokines, triggers the biosynthesis of prostaglandins from the arachidonic acid, which is a 20-carbon polyunsaturated fatty acid released from membrane phospholipids upon the phospholipase A2 action [79]. Worthy, the constitutive expression of COX2 and the sustained biosynthesis of the main effector of inflammation named prostaglandin E2 (PGE-2), are both linked to mammary carcinogenesis due to their stimulatory effects on mitogenesis, invasion and metastasis, angiogenesis and immunosuppression [80–82]. In addition, the overexpression of COX2 may occur in approximately 50% of breast cancer and has been found correlated with a reduced disease-free and overall survival and the primary tumor size [83, 84]. Of note, in line with our findings regarding the hypoxia-dependent activation of the IL-1β/COX2 signaling toward the stimulation of growth and invasion of TNBC cells, previous studies have shown that the MDA-MB-231 cells display a lipidomic profile characterized by high levels of polyunsaturated fatty acids that are known to contribute to breast cancer progression, as mentioned above [85–87].

In the breast cancer microenvironment, the bidirectional interplay occurring among neoplastic cells with CAFs, tumor-associated macrophages, endothelial and immune infiltrating cells, endorses tumor progression stimulating responses as cell proliferation, invasion, angiogenesis and metastasis [18, 19]. In particular, CAFs may facilitate the establishment of a conducive microenvironmental niche for the cruitment and maintenance of disseminated tumor initiating cells [88–90]. Likewise, CAFs may regulate the malignant features fostering the proliferative, migratory and invasive potential of surrounding tumor cells [91]. In this respect, IL-1β was involved in the connection between breast cancer cells and mesenchymal stem cells (MSCs), which are considered an additional source of CAFs beyond the fibroblasts [92, 93]. Worthy, the IL-1β-dictated cellular interaction stimulated the production of diverse chemokines by MSCs as well as an increased motility of TNBC cells [93]. Nicely reminiscing these observations, we have here demonstrated that hypoxic TNBC cells trigger IL-1β/IL1R1 paracrine signals that prompt certain cytoskeletal changes as the MLC-driven formation of actin stress fibers toward invasive properties of breast CAFs. Overall, our data provide novel evidence regarding the mechanisms through which hypoxia triggers the IL-1β/IL1R1 signaling, which generates a feed-forward stimulatory loop in both TNBC cells and CAFs. Our findings may be therefore considered in more comprehensive therapeutic strategies targeting breast cancer progression.

## Conclusions

Our data provide novel evidence regarding the molecular mechanisms through which the IL1β-IL1R signaling does prompt stimulatory effects upon hypoxia in TNBC cells and CAFs. Considering the intricate interplay occurring among diverse players activated by hypoxia even through feed-forward loops, further efforts are warranted to better appreciate this complex scenario toward more comprehensive therapeutic strategies halting the aggressive features of the TNBC.

## Supplementary information

**Additional file 1.** The top 250 genes correlated with HIF-1α in the TCGA dataset were clustered using the KEGG pathway enrichment analysis.

**Additional file 2 **Immunoblots of HIF-1α in MDA-MB-231 cells exposed to IL-1β (10 ng/mL), as indicated. Side panel shows densitometric analysis of the blot normalized to β-actin. Values represent the mean ± SD of three independent experiments performed in triplicate. (*) *p* < 0.05.

**Additional file 3.** Pro-metastatic gene expression changes upon hypoxia or IL-1β in the presence of IL1R1a respect to values set as one-fold induction of cells cultured respectively under normoxia or treated with vehicle.

**Additional file 4 **Immunoblots of IL-1β and COX2 in CAFs exposed to conditioned medium (CM) collected from MDA-MB-231 cells cultured upon normoxia or hypoxia (2% O2), in the presence or absence of a neutralizing IL-1β antibody (140 ng/mL). Side panel shows densitometric analysis of the blots normalized to β-actin. Values represent the mean ± SD of three independent experiments performed in triplicate. (*) *p* < 0.05.

## Data Availability

The datasets generated and/or analyzed during the current study are available in the [https://xenabrowser.net/] and [http://www.cbioportal.org/] repositories.

## Abbreviations

ACTB: Actin beta
CAFs: Cancer-associated fibroblasts
COX2: Cyclooxygenase 2
DAVID: Database for Annotation, Visualization and Integrated Discovery
ECM: Extracellular matrix components
ER: Estrogen receptor
GPCRs: G protein-coupled receptors
GPER: G protein estrogen receptor
GSEA: Gene Set Enrichment Analysis
HER2: Human epidermal growth factor receptor 2
HIF-1α: Hypoxia inducible factor-1α
IL-β: Interleukin-1β
IL1R1: Interleukin 1 Receptor Type 1
IL1R1a: IL1R1 antagonist
KEGG: Kyoto Encyclopedia of Genes and Genomes
METABRIC: Molecular Taxonomy of Breast Cancer International Consortium
NAC: N-acetyl-L-cysteine
PR: Progesterone receptor
ROS: Reactive oxygen species
TCGA: The Cancer Genome Atlas
TNBC: Triple-negative breast cancer
VEGF: Vascular endothelial growth factor
