# Supplementary material for: The IL1β-IL1R signaling is involved in the stimulatory effects triggered by hypoxia in breast cancer cells and cancer-associated fibroblasts (CAFs)
Source: J Exp Clin Cancer Res. 2020 Aug 10;39:153. doi: 10.1186/s13046-020-01667-y (PMC7418191; doi:10.1186/s13046-020-01667-y)
Supplement: Supplementary file 1 — Additional file 1. The top 250 genes correlated with HIF-1α in the TCGA dataset were clustered using the KEGG pathway enrichment analysis. [file 13046_2020_1667_MOESM1_ESM.pdf]

Additional File 1. The top 250 genes correlated with HIF-1α in the TCGA dataset were clustered using the KEGG pathway enrichment analysis. The HIF-1α correlated genes belonging to the "Cytokine-cytokine receptor interaction" pathway were reported according to their correlation coefficients (p-value < 0.001).

**Gene**	**r-value**	**p-value**
OSMR	0.52	7.04E-10
IL1RAP	0.48	1.54E-08
IL-1β	0.44	3.03E-07
IL7R	0.44	3.44E-07
TGFBR2	0.43	5.40E-07
IL1R1	0.43	5.56E-07
IL1A	0.42	1.36E-06
IL6ST	0.41	2.30E-06
INHBA	0.41	2.40E-06
IL6	0.41	2.63E-06
