# Supplementary material for: The IL1β-IL1R signaling is involved in the stimulatory effects triggered by hypoxia in breast cancer cells and cancer-associated fibroblasts (CAFs)
Source: J Exp Clin Cancer Res. 2020 Aug 10;39:153. doi: 10.1186/s13046-020-01667-y (PMC7418191; doi:10.1186/s13046-020-01667-y)

Additional File 2. Immunoblots of HIF-1α in MDA-MB-231 cells exposed to IL-1β (10 ng/mL), as indicated. Side panel shows densitometric analysis of the blot normalized to β-actin. Values represent the mean ± SD of three independent experiments performed in triplicate. (*) $p < 0.05$.

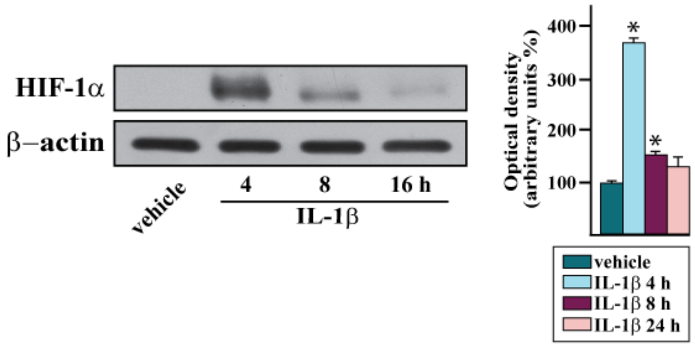

Supplement: Supplementary file 2 — Additional file 2 Immunoblots of HIF-1α in MDA-MB-231 cells exposed to IL-1β (10 ng/mL), as indicated. Side panel shows densitometric analysis of the blot normalized to β-actin. Values represent the mean ± SD of three independent experiments performed in triplicate. (*) p < 0.05. [file 13046_2020_1667_MOESM2_ESM.pdf]
