# Supplementary material for: The IL1β-IL1R signaling is involved in the stimulatory effects triggered by hypoxia in breast cancer cells and cancer-associated fibroblasts (CAFs)
Source: J Exp Clin Cancer Res. 2020 Aug 10;39:153. doi: 10.1186/s13046-020-01667-y (PMC7418191; doi:10.1186/s13046-020-01667-y)
Supplement: Supplementary file 3 — Additional file 3. Pro-metastatic gene expression changes upon hypoxia or IL-1β in the presence of IL1R1a respect to values set as one-fold induction of cells cultured respectively under normoxia or treated with vehicle. [file 13046_2020_1667_MOESM3_ESM.pdf]

Additional File 3. Pro-metastatic gene expression changes upon hypoxia or IL1β in the presence of IL1R1a respect to values set as one-fold induction of cells cultured respectively under normoxia or treated with vehicle.

**Gene**	**Hypoxia (fold changes)**	**IL-1β (fold changes)**
PTGS2 (COX2)	11.38	9.08
IL-1β	10.81	7.16
VEGFA	4.81	1.41
MMP9	4.43	2.92
MMP3	4.34	3.19
SERPINE1	4.16	2.50
TPBG	3.41	1.29
MMP1	2.84	1.46
CD44	2.25	2.40
MYC	2.13	1.46
CTSK	1.70	1.33
